# Plant food consumption and emotional well-being: the Helsinki Health Study among 19–39-year-old employees

**DOI:** 10.1186/s40795-024-00981-4

**Published:** 2024-12-30

**Authors:** Elina Mauramo, Tea Lallukka, Noora Kanerva, Jatta Salmela

**Affiliations:** 1 Department of Public Health, Faculty of Medicine, University of Helsinki, P.O. Box 20, Helsinki, 00014 Finland; 2 Department of Food and Nutrition, Faculty of Agriculture and Forestry, University of Helsinki, Helsinki, Finland

**Keywords:** Plant foods, Emotional well-being, Mental health, Employees, Socioeconomic

## Abstract

**Background and objectives:**

Associations between fruit and vegetable consumption and mental health have been observed, but studies comparing different types of plant foods are sparse. This study among Finnish municipal employees examined associations of the consumption of a range of different plant foods with emotional well-being (EWB).

**Data and methods:**

We used survey data from the Helsinki Health Study conducted in 2017 among 19–39-year-old employees of the City of Helsinki, Finland (N = 5898, response rate 51.5%, 80% women). Consumption of plant foods, including fruit, berries, fresh and cooked vegetables and wholegrain bread, was measured by a food frequency questionnaire and dichotomised into daily/non-daily consumption. The EWB scale of the RAND-36 questionnaire was dichotomised, with the lowest quartile indicating ‘poor EWB’ and the three higher quartiles indicating ‘good EWB’. We used logistic regression for analysing the associations between plant food consumption and EWB. Analyses were sex-stratified and age, socioeconomic circumstances and psychosocial working conditions were adjusted for.

**Results:**

Prevalence of daily consumption of plant foods varied from 25% for berries and cooked vegetables to 70% for fresh vegetables. Daily consumption was associated with good EWB among both women and men. The strongest age-adjusted association was found for fresh vegetables, with women (OR 1.48, 95% CI 1.27–1.74) and men (OR 1.86, CI 1.37–2.52) with daily consumption having clearly higher odds of good EWB compared to non-daily consumers. Associations slightly attenuated but mostly remained after adjusting for socioeconomic circumstances and working conditions.

**Conclusions:**

More frequent plant food consumption was associated with good EWB. Thus, the results support the need for interventions that investigate whether the promotion of plant food consumption could show potential mental health benefits among employees.

## Introduction

Mental health problems of varying degrees are prevalent among employed populations and they greatly contribute to work disability in Finland and elsewhere [1–5]. Thus, to maintain and enhance work ability, it is crucial to identify factors that are linked to better mental health and to find effective ways to support and promote mental well-being in workplaces and in the society in general. In the last decade, there has been a growing interest in the role of diet in mental health. The importance of fruit, vegetable, wholegrain and other plant foods for chronic disease prevention has been known for long [6–8], and lately their associations with different mental health outcomes have been increasingly studied. A high proportion of plant foods in the diet has been suggested to contribute to better outcomes of mental health due to various factors such as their antioxidant and other micronutrient content [9–11].

Most of the previous studies on the associations between plant food consumption and mental health have focused on the consumption of fruit and/or vegetables only. A meta-analysis found that both fruit and vegetable consumption separately as well as combined were associated with a lower risk of depression in cohort and cross-sectional studies [9]. A review of studies among young people and adults aged 15–45 years concluded that a higher fruit consumption level was consistently associated with better mental health in terms of depression and depressive symptoms, but results were less consistent for vegetable consumption [10]. Another review concluded that the consumption of both fresh and processed fruit and/or vegetables as well as some of their specific subgroups is associated with better mental health in adult populations [11]. Some studies focusing on the Mediterranean type of an overall dietary pattern have observed the consumption of grains, legumes and nuts to be associated with better mental health [12]. Evidence from studies including and comparing a wider range of plant foods is, however, still limited. Furthermore, regarding employed populations specifically, evidence is lacking and studies concentrating on different employee groups are warranted to provide information that could be utilised in employee mental health promotion.

The overall level of plant food consumption falls behind from international and national recommendations in high-income countries [6]. In Finland, the most recent national survey showed that only 14% of men and 22% of women reached the minimum recommended consumption level, 500 g per day, of vegetables, fruit and berries [13]. In particular, plant food consumption has been shown to be less frequent among people in lower socioeconomic positions, including lower occupational class, education and income, compared to people in higher positions [14–18]. In addition, among employed populations, work-related factors such as shift work and work stress have been shown to be associated with overall unhealthier food habits and dietary patterns [19, 20]. Thus, it is important to consider socioeconomic as well as work-related factors when inspecting associations between plant food consumption and mental health outcomes.

In this study among municipal employees from Helsinki, the capital of Finland, we examined whether the consumption frequency of different plant foods is associated with emotional well-being (EWB). The examined plant foods included fruit, berries, fresh and cooked vegetables and wholegrain bread. In addition, we examined whether socioeconomic circumstances or psychosocial working conditions contributed to the associations between plant food consumption and EWB. The examined socioeconomic circumstances included education, occupational class, household income and current economic difficulties, and the psychosocial working conditions consisted of working time, shift work, mental workload, workplace atmosphere and bullying.

## Data and methods

### Survey data

**Fig. 1:**
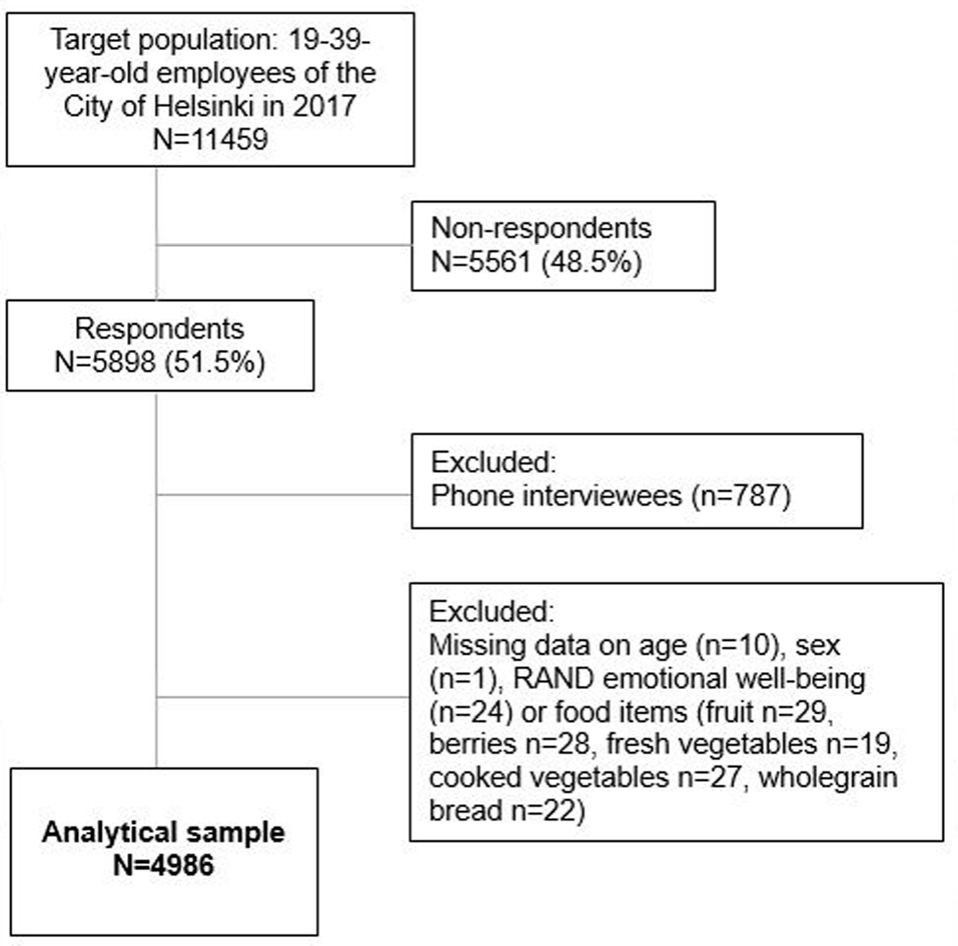
Flow chart of the selection of the analytical sample of the Helsinki Health Study participants in 2017

We used survey data from the Helsinki Health Study (HHS) among 19–39-year-old employees. The survey was conducted in 2017 among all those employees of the City of Helsinki, Finland, who were born in 1978 or later, and who at the time of the survey had been employed for 4 months or more with an employment contract of at least 50% [21]. The survey was conducted using (1) online questionnaires, (2) practically identical postal questionnaires among those who did not have an email address at work (however, it was possible to choose an online survey using a personal link provided with the mailed questionnaire), and (3) telephone interviews among those who did not respond to the online or postal questionnaires. Altogether 5898 employees responded (response rate 51.5%). Of the respondents, 78.5% were women which corresponds to the sex distribution among the employees of the City of Helsinki. Since the telephone interviews were notably shortened and did not include most of the measures used in this study, we excluded telephone interviewees. The final number of participants who had full information on the plant food items and EWB was 4986 (80% women). The flow chart of the sample selection is shown in Fig. 1. The ethical aspects of the Helsinki Health Study have been approved by the ethics committee at the Faculty of Medicine, University of Helsinki, and the City of Helsinki health authorities.

### Food frequency questionnaire

Consumption of different food items was measured with a 14-item food frequency questionnaire (FFQ). In this study, the utilised food items of the FFQ consisted of fruit, berries, fresh vegetables, cooked vegetables and wholegrain bread. These foods of plant origin have generally been considered as indicating an overall healthier dietary pattern [6, 7, 22]. Respondents were asked to estimate their overall consumption frequency of the different food items during the past 4 weeks, with the following frequency categories: ‘not during the past 4 weeks’, ‘1–3 times a month’, ‘once a week’, ‘2–4 times a week’, ‘5–6 times a week’, ‘once a day’ and ‘two times or more daily’. For each category, we calculated the average frequency per day and then multiplied by 28 days to produce the total number of consumption times per four weeks: 0, 2, 4, 12, 22, 28 and 56, following our previous studies [4, 23]. In addition, we formed a dichotomous variable of daily versus non-daily consumption of each food item. At least 28 consumption times per four weeks was considered as daily consumption and less than that as non-daily consumption.

### Emotional well-being

EWB was measured with the emotional well-being scale of the RAND-36 questionnaire which is a reliable and well-validated self-report health survey instrument [24]. EWB scale was based on five questions concerning the past four weeks preceding the survey. The questions inquired how much of the time the respondent had been very nervous, had felt so down that nothing could cheer him/her up, had felt calm and peaceful, had felt downhearted and blue and had been a happy person. The six-point response scale ranged from 1 ‘all the time’ to 6 ‘none of the time’. We calculated a total sum score, ranging from 0 to 100. A participant received a score value if responding to any of the five questions. There were altogether 67 participants who had a missing value in one of the five questions, but none of the participants had multiple missing values. To be able to compare participants with lower and higher scores, we dichotomised the measure [25], using 60 as the cut-off point based on previous literature [26, 27], into ‘poor’ (sum score ≤ 60) and ‘good’ (sum score > 60) EWB.

### Covariates

Covariates included age, sex, socioeconomic circumstances and psychosocial working conditions. We divided education into three levels: high (master’s degree or higher), intermediate (bachelor’s degree) and low (upper secondary school or lower), according to previous procedures [27]. Occupational class consisted of four hierarchical categories: managerial and professional (e.g. teachers and physicians), semi-professional (e.g. nurses and foremen), routine non-manual employees (e.g. childcare and elderly care workers) and manual workers (e.g. care assistants). The information was derived from the personnel register data for those who consented to register linkage (82%) and was completed from the survey data for the rest. Household income was based on a question asking about the total typical monthly income of the respondent’s household. We divided the monthly income by household size and weighted according to the modified OECD equivalence scale which means that the respondent received the value of 1.0, other adults 0.5 and children 0.3 [28]. We formed four hierarchical income groups with each of them consisting of approximately a quarter of the study population. Current economic difficulties were measured with two questions from Pearlin’s list of chronic strains: [29] ‘How much difficulty do you have in meeting the payment of bills?’ and ‘How often do you have enough money to buy the food or clothing you or your family need?’. Five response alternatives indicated the level of difficulties: ‘very little’ to ‘very great’ for the first question, and ‘always’ to ‘never’ for the second question. A combined variable was formed and categorised into no, occasional and frequent difficulties.

Psychosocial working conditions included (1) working time of 40 + hours per week versus less, (2) shift work versus normal working hours, (3) mental workload measured with a single question asking how heavy or light the respondent considered the work to be (very light / rather light / moderately heavy / very heavy), (4) workplace atmosphere measured with a single question enquiring about the atmosphere and categorised into good and poor, and (5) workplace bullying measured with an instructed question about being bullied currently, previously or never.

### Statistical analyses

First, we calculated descriptive numbers and percentages. Next, we calculated the distribution of good/poor EWB by daily/non-daily consumption of the plant foods with p-values from the Pearson’s chi-square test. After these descriptive analyses, we fitted logistic regression models producing odds ratios (OR) with their 95% confidence intervals (CI) to examine associations between the daily/non-daily consumption of plant foods and good/poor EWB. We fitted models separately for women and men due to previously observed differences in food consumption [23]. First, we fitted age-adjusted base models (Model 1). Then we adjusted for socioeconomic circumstances (Models 2 and 3) and working conditions (Models 4 and 5) in the following models. We performed the analyses using SAS statistical software version 9.4 (SAS Institute Inc., Cary, NC, USA).

## Results

The prevalence of daily consumption among the participants varied between the different plant foods. The lowest prevalence percentage of daily consumption, 25%, was found for berries and cooked vegetables and the highest percentage,70%, for fresh vegetables (Table 1). The mean scores of EWB were higher among participants with daily consumption of each of the plant foods compared to those with non-daily consumption (Table 1).

Among women, participants reporting daily consumption of fruit, berries, fresh and cooked vegetables and wholegrain bread had in general higher prevalence of good EWB compared to participants reporting non-daily consumption (Table 2). The largest difference was found for fresh vegetables, with 78% of daily consumers having good EWB compared to 70% of non-daily consumers (*p* < 0.001). Among men, the prevalence percentages of having good EWB were broadly similar to women. The largest difference was found for fresh vegetables, with 83% of daily and 72% of non-daily consumers having good EWB (*p* < 0.001).

**Table 1:** Distribution (N, %) of participants and RAND-36 emotional well-being (EWB) score means with standard deviation (SD)

		*N*	%	EWB score, mean (SD)
**Plant food consumption**				
Fruit	Daily	2351	47	74.2 (16.2)
	Non-daily	2635	53	71.1 (17.5)
Berries	Daily	1260	25	74.3 (16.8)
	Non-daily	3726	75	72.0 (17.0)
Fresh vegetables	Daily	3466	70	73.8 (16.1)
	Non-daily	1520	30	69.7 (18.4)
Cooked vegetables	Daily	1263	25	74.0 (16.4)
	Non-daily	3723	75	72.1 (17.1)
Wholegrain bread	Daily	2003	40	74.0 (16.5)
	Non-daily	2983	60	71.6 (17.2)
**Sex**	Women	3983	80	72.3 (17.0)
	Men	1003	20	73.5 (16.7)
**Age**	19–29	1577	32	71.2 (17.8)
	30–39	3409	68	73.2 (16.5)
**Education**	High	1459	29	73.0 (15.7)
	Intermediate	1819	37	73.2 (16.8)
	Low	1698	34	71.4 (18.1)
**Occupational class**	Professional	1352	27	73.0 (15.9)
	Semi-professional	1984	40	73.0 (16.8)
	Routine non-manual	1354	27	72.2 (17.5)
	Manual worker	265	5	70.0 (20.3)
**Household income**	Highest	949	19	74.8 (15.5)
	2nd	1220	25	74.3 (15.5)
	3rd	1132	23	71.2 (17.4)
	Lowest	1653	33	70.9 (18.1)
**Current economic difficulties**	No	2244	45	75.8 (15.4)
	Occasional	2280	46	71.3 (17.0)
	Frequent	452	9	62.7 (19.7)
**Working time**	Less than 40 h/week	4058	81	72.6 (16.9)
	40 h or more /week	928	19	72.2 (17.2)
**Shift work**	No	3461	71	72.8 (16.7)
	Yes	1401	29	71.8 (17.7)
**Mental workload**	Low	4061	82	74.3 (15.9)
	High	881	18	64.4 (19.1)
**Being bullied at workplace**	No	3225	65	75.0 (15.7)
	Yes, in previous workplace	1014	21	68.6 (17.7)
	Yes, in current workplace	693	14	67.3 (19.0)
**Workplace atmosphere**	Good	3559	72	74.6 (15.8)
	Less than good	1388	28	67.3 (18.7)
**All**		4986	100	72.6 (17.0)

**Table 2:** Emotional well-being (EWB, good/poor; n,%) by plant food item consumption (daily/non-daily) among women and men

	Women			Men		
	Good EWB	Poor EWB	*P*-values	Good EWB	Poor EWB	*P*-values
	*N*, %	*N*,%		*N*,%	*N*,%	
**Fruit**			< 0.001			0.003
Daily	1614 (78.9)	433 (21.2)		255 (83.9)	49 (16.1)	
Non-daily	1397 (72.2)	539 (27.8)		526 (75.3)	173 (24.7)	
**Berries**			0.003			
Daily	892 (78.9)	239 (21.1)		107 (83.0)	22 (17.1)	0.137
Non-daily	2119 (74.3)	733 (25.7)		674 (77.1)	200 (22.9)	
**Fresh vegetables**			< 0.001			
Daily	2283 (77.7)	654 (22.3)		439 (83.0)	90 (17.0)	< 0.001
Non-daily	728 (69.6)	384 (30.4)		342 (72.2)	132 (27.9)	
**Cooked vegetables**			0.006			0.046
Daily	870 (78.6)	237 (21.4)		131 (84.0)	25 (16.0)	
Non-daily	2141 (74.4)	735 (25.6)		650 (76.7)	197 (23.3)	
**Wholegrain bread**			0.02			0.041
Daily	1284 (77.5)	372 (22.5)		283 (81.6)	64 (18.4)	
Non-daily	1727 (74.2)	600 (25.8)		498 (75.9)	158 (24.1)	

The logistic regression analyses showed clear associations between all of the plant food items and EWB among women (Table 3). Daily consumption of fruit, berries, fresh vegetables, cooked vegetables, and wholegrain bread was associated with good EWB (Model 1, age-adjusted OR range from 1.16 to 1.48). The strongest associations were found for fresh vegetables and fruit. Participants with daily consumption of fresh vegetables had clearly higher odds for having good EWB in comparison with non-daily consumers (Model 1, age-adjusted OR 1.48, 95% CI 1.27–1.74), similarly to participants with daily consumption of fruit (Model 1, age-adjusted OR 1.42, 95% CI 1.23–1.64). After adjustments for socioeconomic circumstances, especially for household income and current economic difficulties, in Models 2 and 3, most of the associations were attenuated to some extent (Table 3). Adjustments for working conditions, in Models 4 and 5, had only negligible or minor contributions to the associations (Table 3).

**Table 3:** Plant food consumption and good emotional well-being among women. Odds ratios with 95% confidence intervals from logistic regression

	MODEL 1 (M1):	MODEL 2:	MODEL 3:	MODEL 4:	MODEL 5:
	Age-adjusted	M1 + education, occupational class	M1 + household income, economic difficulties	M1 + working time, shift work	M1 + mental workload, being bullied at workplace, workplace atmosphere
**Daily consumption of (ref. non-daily)**					
Fruit	1.42 (1.23–1.64)	1.39 (1.20–1.61)	1.32 (1.14–1.54)	1.42 (1.23–1.65)	1.38 (1.19–1.61)
Berries	1.28 (1.09–1.51)	1.25 (1.06–1.48)	1.22 (1.03–1.45)	1.28 (1.08–1.51)	1.27 (1.07–1.52)
Fresh vegetables	1.48 (1.27–1.74)	1.45 (1.23–1.70)	1.38 (1.17–1.62)	1.49 (1.27–1.76)	1.46 (1.23–1.72)
Cooked vegetables	1.23 (1.04–1.45)	1.20 (1.01–1.42)	1.17 (0.99–1.39)	1.24 (1.04–1.47)	1.25 (1.05–1.49)
Wholegrain bread	1.16 (1.00-1.35)	1.14 (0.98–1.32)	1.14 (0.98–1.32)	1.14 (0.98–1.33)	1.09 (0.93–1.28)

Among men, similar associations were found with even higher ORs, although with wider CIs, than among women (Table 4). Men with daily consumption of fruit, berries, fresh and cooked vegetables, and wholegrain bread had higher odds of good EWB compared to men with non-daily consumption (Model 1, age-adjusted OR range from 1.38 to 1.86). However, for berries and cooked vegetables the association was not statistically significant. The strongest association was, similarly to women, found for fresh vegetables, with daily consumers having higher odds for good EWB compared to non-daily consumers (Model 1, age-adjusted OR 1.86, 95% CI 1.37–2.52). After adjustments for socioeconomic circumstances, especially for household income and current economic difficulties, in Models 2 and 3, most of the associations were slightly attenuated (Table 4). Similarly to women, adjustment for working conditions, in Models 4 and 5, had negligible or minor contributions to the associations (Table 4).

**Table 4:** Plant food consumption and good emotional well-being among men. Odds ratios with 95% confidence intervals from logistic regression

	MODEL 1 (M1):	MODEL 2:	MODEL 3:	MODEL 4:	MODEL 5:
	Age-adjusted	M1 + education, occupational class	M1 + household income, economic difficulties	M1 + working time, shift work	M1 + mental workload, being bullied at workplace, workplace atmosphere
**Daily consumption of (ref. non-daily)**					
Fruit	1.69 (1.19–2.40)	1.60 (1.12–2.28)	1.64 (1.14–2.35)	1.68 (1.18–2.40)	1.66 (1.15–2.41)
Berries	1.44 (0.89–2.35)	1.41 (0.86–2.30)	1.39 (0.84–2.28)	1.40 (0.86–2.29)	1.39 (0.83–2.32)
Fresh vegetables	1.86 (1.37–2.52)	1.70 (1.24–2.32)	1.66 (1.21–2.28)	1.91 (1.40–2.60)	1.80 (1.30–2.48)
Cooked vegetables	1.56 (0.98–2.46)	1.49 (0.93–2.39)	1.43 (0.89–2.29)	1.58 (0.99–2.51)	1.45 (0.90–2.35)
Wholegrain bread	1.38 (0.99–1.91)	1.41 (1.01–1.96)	1.35 (0.96–1.90)	1.39 (1.00-1.94)	1.24 (0.88–1.75)

## Discussion

This study examined associations between the consumption frequency of plant foods and EWB among 19–39-year-old employees of the City of Helsinki, Finland. The main finding of the study was that more frequent consumption of most of the plant food items was associated with better EWB. Overall, the strongest association was found between the consumption of fresh vegetables and EWB among both women and men. Associations remained after adjusting for socioeconomic circumstances and working conditions, although household income and current economic difficulties attenuated the associations modestly.

Our results are in line with previous studies, conducted among different populations and in varying settings, which have suggested a higher level of fruit and vegetable consumption to contribute to better mental health outcomes. A meta-analysis of eighteen studies found that both fruit and vegetable intake separately as well as combined were associated with a lower risk of depression in cohort and cross-sectional studies [9]. A Canadian longitudinal study showed fruit and vegetable consumption to be inversely associated with later depression and psychological distress in two-year cycles [30]. A recent prospective study among young Australian women found a higher fruit (≥ 4 servings) and vegetable (≥ 5 servings) intake to be associated with lower odds of depressive symptoms in comparison to one serving or less per day [31]. The previous studies have, however, not focused especially on employees but rather examined general adult populations. Thus, our results provide novel insights examining an employee population, that is, a population from which the poorest and least healthy have been left out due to the so-called healthy worker effect [32].

In this study, all of the examined plant foods showed some positive associations with EWB. The strongest associations were found for fresh vegetables among both women and men and also for fruit among women. Compared to many of the earlier studies, which have mostly examined fresh vegetable and fruit consumption only, this study included a slightly wider range of plant foods. We examined the different food items separately–instead of total consumption–to detect possible differences. There are some earlier results on differences between various categories of plant foods. A small survey among young adults from the United States and New Zealand found raw fruit and vegetables to be associated with reduced depressive symptoms and more positive mood, while fruit and vegetables that were cooked or canned did not show such associations [33]. A study on the American NHANES data found that besides total fruit and vegetable intake, the consumption of berries, tomatoes, green vegetables and dried fruit showed inverse associations with depressive symptoms [34]. Wholegrain consumption, which showed modest associations with EWB in this study, has seldom been studied in relation to mental health, but some earlier evidence exists. Among Chinese adults a higher consumption level of wholegrain foods was associated with a lower level of depressive symptoms [35]. A review study suggested that a diet high in fiber from wholegrains among other plant foods could benefit mental health [36]. It should be noted that in our study, information was available concerning wholegrain bread only, and thus, other wholegrain foods, such as porridge or brown rice, were not covered.

This study examined the consumption frequency as categorised into daily versus non-daily consumption. It should be noted that this is a broad measure of frequency and does not include information on the quantity of consumption. Direct comparisons cannot thus be made with the national and international dietary recommendations, but it is likely that even among participants in the “good” category, that is daily use, the consumption is to a large part lower than what is recommended in Finland and internationally [13]. However, studies with different kinds of dietary assessment methods have produced largely parallel results on the associations between dietary factors and mental health. Concerning plant foods, previous studies have found associations with mental health outcomes utilising varying frequency or quantity measures capturing shorter or longer term food consumption. In a Swiss study, a recommendation of “5-a-day” was used and consuming the five portions of vegetables and fruit per day was associated with better mental health indicated by lower psychological distress [37]. An Iranian study converted consumption frequencies reported by participants into a quantity in grams per day, and found the highest consumption level, compared to the lowest, to be associated with lower odds of depression and distress [38]. A Canadian longitudinal study utilised a daily fruit and vegetable consumption score which showed inverse associations with depressive symptoms [30]. Overall, our results confirm the findings of the previous studies, which have, regardless of the exact measurements and methods, shown positive associations with mental health and well-being for even a relatively low level of plant food consumption.

We examined women and men separately due to previous studies having shown sex differences in both plant food consumption and mental health [23, 39, 40]. With regard to the associations between plant food consumption and mental health outcomes, very few studies have previously considered differences between women and men. Among Iranian adults, women but not men with high intake of fruit and vegetables were found to have lower odds of depression [38], whereas among Swiss general population, sex differences were not found for associations between fruit and vegetable consumption and psychological distress [37]. In this study, we found fewer associations to be statistically significant among men than women. This difference could be due to the smaller number of men in the study which likely affects the statistical power. However, the associations between plant food consumption and EWB had the same direction, and the differences in the estimates that were observed between daily and non-daily consumers of the various food items were mostly larger among men than among women.

In this study, adjusting for socioeconomic circumstances, and to a lesser degree also for working conditions, had some attenuating effects on the observed associations between plant food consumption and EWB. Especially, adjustments for household income and current economic difficulties affected the associations. Previous studies examining associations between plant food consumption and mental health have mostly considered only single socioeconomic factors, mainly education, and the findings have been inconsistent [9, 37, 41]. However, many earlier studies have shown that a lower socioeconomic position and more disadvantageous socioeconomic circumstances are associated with a lower level of plant food consumption [14, 16–18]. In our recent study, especially lower income and current economic difficulties showed clear associations with belonging to lower long-term fruit and vegetable consumption trajectories among ageing employees [14]. Based on the results of this study, further, more detailed studies on the mechanisms of the associations among different employee groups are clearly warranted.

Overall, in the light of previous evidence, it could be suggested that the results of this study, in their part, confirm the importance of plant food consumption, highlighting the need for intervention studies related to the dietary habits and mental health among employees. Since mental health problems have been shown to be frequent among employees, it would be crucial to develop efficient measures and policies to promote employees’ mental well-being in the long term. Increasing fruit, vegetable and other plant food consumption could have such potential, and different kinds of workplace intervention studies on the ways to increase plant food use have already been conducted internationally [42–44]. The potential benefits of an increased plant food consumption are also supported by trial studies which have shown that a dietary pattern with a high level of various plant foods could have positive influence and even therapeutic effects on mental health [11, 45, 46]. However, further intervention studies are warranted in order to obtain information concerning mental health specifically as well as to find cost-effective ways to implement the information among different employee groups and working environments.

In addition to interventions, longitudinal studies with follow-up data over a longer time period would provide the most useful information. Future studies should also consider register-based mental health outcomes in particular, as well as other employee groups including municipal employees from different age groups as well as employees elsewhere in the public and private sectors. Specifically, employees in manual work would be a group in which there could be the largest potential in terms of increasing the overall consumption level of plant foods as a way to promote employee mental health and work ability.

### Methodological considerations

This cross-sectional study was based on survey data among young and midlife municipal employees. According to a non-response analysis, the study population can be considered as representative of the target population, the female and male 19- to 39-year-old employees of the City of Helsinki, Finland [21]. The proportion of female employees was high among the respondents, 78.5%, corresponding to the overall sex distribution among the employees of the City of Helsinki. In general, the results are, with caution, generalisable to young and midlife employees in the municipal sector in Finland, and possibly also elsewhere in the public sector. To some degree, the results might also be generalisable to similar employee populations in other high-income countries, although caution should be applied due to possible cultural differences in, for example, food habits. With regard to the measure of food consumption, the 14-item FFQ, short FFQs have in previous studies been shown to be suitable as measures of frequently consumed foods [47].

The limitations include the characteristics of the survey data and the study population consisting of municipal employees which should be taken into account in the interpretation of the results and when making generalisations. In Finland, a large proportion of the municipal employees are employed in education, social and healthcare sectors, due to which the majority of both the target population, the City of Helsinki employees, and the study participants were women [21]. The lower number of male participants causes restrictions to the statistical power among men in our data. The data also included participants who were not working at the time of the survey due to for example parental or sick leave (*N* = 512). Therefore, we tested whether adjusting for the current employment status would affect the results and found negligible effects (no data shown). With regard to the EWB measurement, it should be noted that the category of ‘poor well-being’ was based on the lowest quartile of the score and the outcome is thus relative, comparing participants with lower and higher scores. The survey measures in general cause limitations. The FFQ was not validated and it did not include portion sizes or information on whether the consumed items were part of a main meal or snacks, for example. Consumption quantities could thus not be assessed. It has been shown, however, that the contribution of portion size questions in FFQs to the food intake variance may be negligible [48]. In addition, the general cautions warranted when interpreting results obtained with self-reported data, due to, for example, under- or over-reporting, apply to this study. Especially, over-reporting is a possibility with regard to the consumption of plant foods. Also, it should be noted that causality cannot be determined with cross-sectional survey data. Reverse associations are also possible, that is, poorer mental health could influence food habits, leading to a lower level of consumption of plant foods [11, 49].

## Conclusions

This study among Finnish municipal employees found more frequent, that is, daily versus non-daily, plant food consumption to be associated with good EWB. The results of this study warrant interventions that investigate whether the promotion of plant food consumption could show potential mental health benefits among employees.

## Data Availability

The data are kept at the University of Helsinki protected computers and are available for research purposes upon agreement with the Helsinki Health Study.
